# 

**Published:** 1986-06

**Authors:** 


					MEDICO
CHIRURGICAL
JOURNAL
VOLUME 101(iii)
JUNE 1986
The History of Mental Handicap in Bristol and Bath, p 53
Witchcraft in Post-colonial Africa, p 64
Gardening?The Great Test of Health. p66
News and Comment, p 60.
And Much Else
IN ASSOCIATION WITH
BRISTOL MEDICINE
THE MEDICAL JOURNAL OF THE SOUTH WEST

				

